# JAK2 V617F Constitutive Activation Requires JH2 Residue F595: A Pseudokinase Domain Target for Specific Inhibitors

**DOI:** 10.1371/journal.pone.0011157

**Published:** 2010-06-16

**Authors:** Alexandra Dusa, Céline Mouton, Christian Pecquet, Murielle Herman, Stefan N. Constantinescu

**Affiliations:** 1 Ludwig Institute for Cancer Research Ltd., Brussels, Belgium; 2 de Duve Institute, Université catholique de Louvain, Brussels, Belgium; Institut Européen de Chimie et Biologie, France

## Abstract

The JAK2 V617F mutation present in over 95% of Polycythemia Vera patients and in 50% of Essential Thrombocythemia and Primary Myelofibrosis patients renders the kinase constitutively active. In the absence of a three-dimensional structure for the full-length protein, the mechanism of activation of JAK2 V617F has remained elusive. In this study, we used functional mutagenesis to investigate the involvement of the JH2 αC helix in the constitutive activation of JAK2 V617F. We show that residue F595, located in the middle of the αC helix of JH2, is indispensable for the constitutive activity of JAK2 V617F. Mutation of F595 to Ala, Lys, Val or Ile significantly decreases the constitutive activity of JAK2 V617F, but F595W and F595Y are able to restore it, implying an aromaticity requirement at position 595. Substitution of F595 to Ala was also able to decrease the constitutive activity of two other JAK2 mutants, T875N and R683G, as well as JAK2 K539L, albeit to a lower extent. In contrast, the F595 mutants are activated by erythropoietin-bound EpoR. We also explored the relationship between the dimeric conformation of EpoR and several JAK2 mutants. Since residue F595 is crucial to the constitutive activation of JAK2 V617F but not to initiation of JAK2 activation by cytokines, we suggest that small molecules that target the region around this residue might specifically block oncogenic JAK2 and spare JAK2 wild-type.

## Introduction

JAK2 belongs to the Janus kinases (JAKs) family of non-receptor tyrosine kinases, crucial to blood formation and immune responses. JAK2 plays a role in downstream signaling pathways such as the JAK/STAT pathway, involved in cytokine signaling. Members of the JAK family possess seven defined regions of conserved homology denoted JAK homology (JH) domains 1-7 [1]. JH5-7 make up the amino terminus of JAKs and contain a predicted FERM (Band-4.1, ezrin, radixin and moesin)-like motif [2], important in association of JAKs to their receptors and in some cases in receptor cell-surface expression [3], [4], [5]. Although the JH3-4 domains display some homology to SH2 domains, their function remains ambiguous [6]. The carboxyl terminus is composed of JH1 and JH2 and contains the kinase and pseudokinase domains, respectively [7]. The JAK2 JH1 domain includes all the features of a catalytic tyrosine kinase, while the JH2 domain, though highly sequence-homologous to JH1, lacks several elements conferring catalytic activity. Interestingly, early functional studies showed an inhibitory effect of pseudokinase domain on the kinase domain of JAK2 [8], [9].

While currently there is no three-dimensional structure for any full-length Janus kinase, the crystal structures of JAK2, JAK1 and JAK3 kinase domains in isolation have been solved in complex with specific inhibitors [10], [11], [12]. The JAK2 kinase domain exhibits a typical bilobar arrangement, with a secondary structure profile very similar to other solved kinase domains [13], [14]. The N-terminal lobe of JAK2 is composed of β-strands and includes a single helix, αC, while the C-terminal lobe is mostly helical [10], [12].

A single acquired somatic mutation in the pseudokinase domain of JAK2, in the form of a substitution of Val for Phe at position 617, is at the base of >95% Polycythemia Vera (PV) patients and 50–60% of patients with Essential Thrombocythemia (ET) and Primitive Myelofibrosis (PMF) [15], [16], [17], [18]. The V617F mutation induces constitutive tyrosine phosphorylation of JAK2 and STAT5 and renders Ba/F3 cells that express the erythropoietin receptor (EpoR) cytokine-independent.

Despite a plethora of recent reports describing the contribution of V617F to different pathologies, a comprehensive mechanism of activation of this mutation has yet to be proposed. In this work we explore the role of the pseudokinase domain helix C in the constitutive activation of JAK2 V617F by focusing on residue F595, predicted to be located in the middle of the helix.

## Results

### A predicted interaction between residue 617 and the JH2 αC helix

The structure of a complex of the kinase (JH1) and pseudokinase (JH2) domains has not been solved for any JAK family member. Since residue V617 is located in the pseudokinase domain of JAK2, this lack of information has hindered a detailed understanding of the mechanism of activation of JAK2 V617F. A homology model of the kinase and pseudokinase domains of JAK2, suggests an overall 3D structure of the JH2 domain, similar to that of JH1 and other solved kinase domains, as well as a potential face-to-face arrangement of the two domains [19]. This model places residue V617 in a loop connecting β-strands 4 and 5 in the N-terminal lobe of JH2 and in close proximity to the JH2 αC helix. The β4/β5 loop as well as the αC-β4 loop that precedes β4, were previously shown to play regulatory roles in the mechanisms of Src and Abl tyrosine kinases through interactions with the kinase domain αC helix in the N-terminal lobe [20], [21]. A specific conformation of the kinase domain αC helix is essential for kinase activation [22] and members of the kinase family have evolved diverse ways to influence the position of their αC helices as a means to affect activity [13], [14], [23].

**Figure 1:**
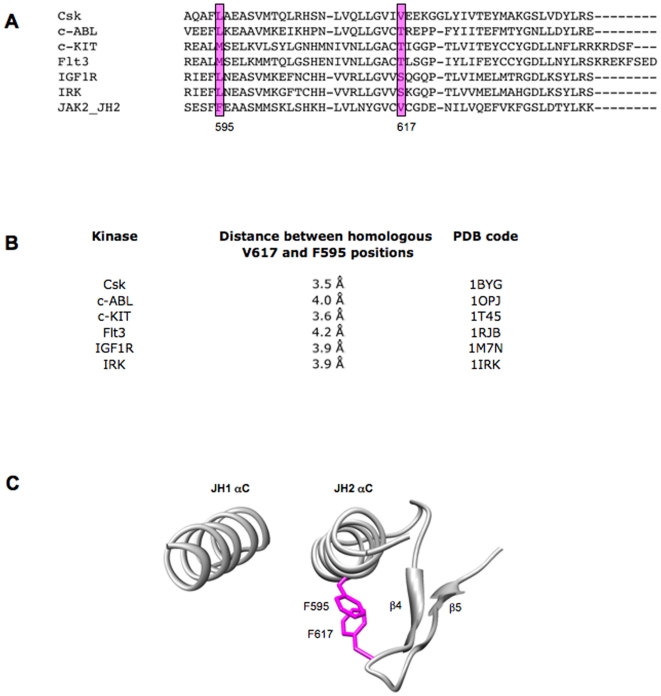
Sequence homology and distances between homologous JAK2 V617 and F595 positions in other kinases and a model of the relative positions of the two residues in JAK2. (A) Alignment of part of the pseudokinase domain of JAK2 with other kinases containing a GVCV-like motif. (B) Distances between the nearest atoms of homologous F595 and V617 residues in the other kinases. Distances were estimated based on the PDB coordinates with UCSF Chimera program [46]. (C) Predicted relative position of the JAK2 JH1 and JH2 αC helices based on the homology model of Lindauer et al [19]. In this predicted arrangement, residue F595 in the JH2 αC helix is in the proximity of mutated V617F residue, also located in the pseudokinase domain.

Given the proximity of the V617F mutation to the JH2 αC helix, we sought to identify residues in the JH2 αC helix, which could have a potential effect on the overall position and conformation of this helix. Inspection of the crystal structures of several kinases revealed that the closest residue in space to the homologous V617 position frequently resided in the middle of the αC helix, on the face of the helix facing the homologous V617 residue (Figure 1A and B). In JAK2, this residue corresponds to F595 in the JH2 αC helix (Figure 1C). The homologous residues of position 595 are generally represented by large, hydrophobic residues pointing to preservation of the hydropathy profile at this position, and suggesting a mechanistic role for those residues in kinase function (Figure 1A).

### Constitutive activity of JAK2 V617F requires F595

We hypothesized that when V617 is mutated to Phe, this could influence the neighboring F595, affecting the conformation of the αC helix of JH2 in a specific manner, that would promote activation in the absence of ligand (Figure 1C). Such Phe-Phe stacking interactions in regulatory domains were shown to play a role in the regulation of catalysis of c-Src [24] and ZAP-70 kinases [25].

**Figure 2:**
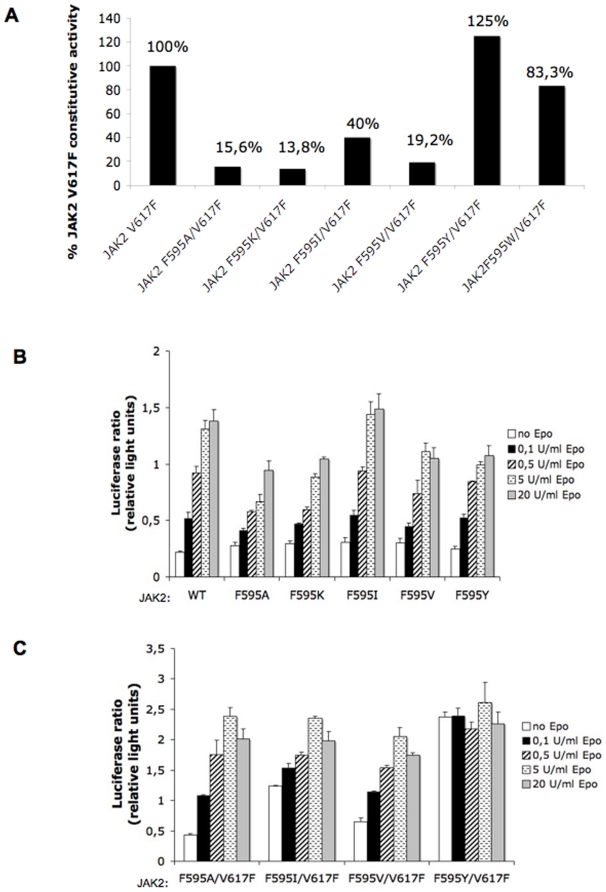
Effect of the substitution of residue F595 in the JH2 αC helix on the constitutive activity of JAK2 V617F and on the ligand-induced activation of JAK2 wild-type measured by luciferase assays. (A) Mutation of F595 to some residues induces up to an 86% decrease in the constitutive activity of JAK2 V617F, depending on the particular substitution, while mutation of F595 to aromatic residues rescues the constitutive activity of JAK2 V617F. The activity of wild-type JAK2 and of JAK2 F595A were similar and amounted to 15-20% of the activity of JAK2 V617F (not shown). (B) The single substitution mutants at position 595 display Epo responses similar to JAK2 wild-type at various concentrations of Epo and do not induce constitutive activation of JAK2 wild-type. (C) The JAK2 F595X/V617F double mutants also respond to Epo in a manner analogous to JAK2 wild-type.

We have previously shown that mutation of V617 to Ala does not support constitutive activation of JAK2 [26]. In order to further investigate the effect of the predicted contact between F595 and F617, we mutated the Phe at position 595 of murine JAK2 to several different amino acids in the context of V617F, and tested the activity of the double mutants in luciferase assays by quantifying the STAT5 trancriptional activity induced by each mutant in transient transfections in the JAK2-deficient fibrosarcoma cell line, γ-2A [27]. As the γ-2A cell line does not express any endogenous JAK2, the activity levels we elicited were due solely to our transfected mutants. We observed up to an 85% decrease in the constitutive activity of JAK2 V617F, depending on the particular amino acid substitution at position 595 (Figure 2A). Mutation of F595 to Lys elicited the highest decrease in JAK2 V617F constitutive activity, followed by Ala, Val and Ile. Contrarily, mutation of F595 to Tyr or Trp, supported a level of constitutive activity of V617F similar to non-mutated F595, indicating the presence of an aromatic residue at position 595 is required to maintain the constitutive activity of JAK2 V617F (Figure 2A).

Next, we asked whether mutation of the F595 residue to Ala, Lys, Val or Ile critically influenced the ability of wild-type JAK2 or of JAK2 V617F to respond to Epo-activated EpoR. As depicted in Figure 2B, F595 mutations generally do not impair activation by Epo. The F595I mutant responds to Epo indistinguishably from JAK2 wild-type. Mutants F595A and F595V exhibit a small inhibition to high Epo doses (Figure 2B), while in the V617F context these mutants do not affect Epo response (Figure 2C). The F595K mutants in contrast, appear to reduce Epo activation of JAK2 V617F to a certain degree (data not shown). Importantly, the single mutations at F595 did not induce constitutive activation of JAK2 (Figure 2B). Taken together, our results indicate that helix C residue F595 is critical for constitutive activation of JAK2 signaling by V617F, and that the nature of the amino acid substitution at F595 can alter the full response to ligand but is not required for initiation of activation of JAK2 in response to cytokine receptor activation.

Interestingly, mutating the beginning segment of the JH2 αC helix did not have the same effect on the constitutive activity of JAK2 V617F as mutating F595. We introduced a R588A/E592A mutation in JAK2 V617F and observed no significant decrease in STAT5 transcriptional activity relative to the nonmutated protein, unless F595A was additionally introduced (Figure S1). Assuming a regular alpha helical pattern for the JH2 αC helix, residues 588 and 592 would be located on the same face of the helix as F595, each on one of the preceding turns. The fact that they do not alter the constitutive activity of JAK V617F suggests that F595 plays a specific role in supporting activation by the V617F mutation.

### Functional studies of the F595A mutation on the proliferation and transformation of Ba/F3 cells stably expressing JAK2 F595A/V617F

**Figure 3:**
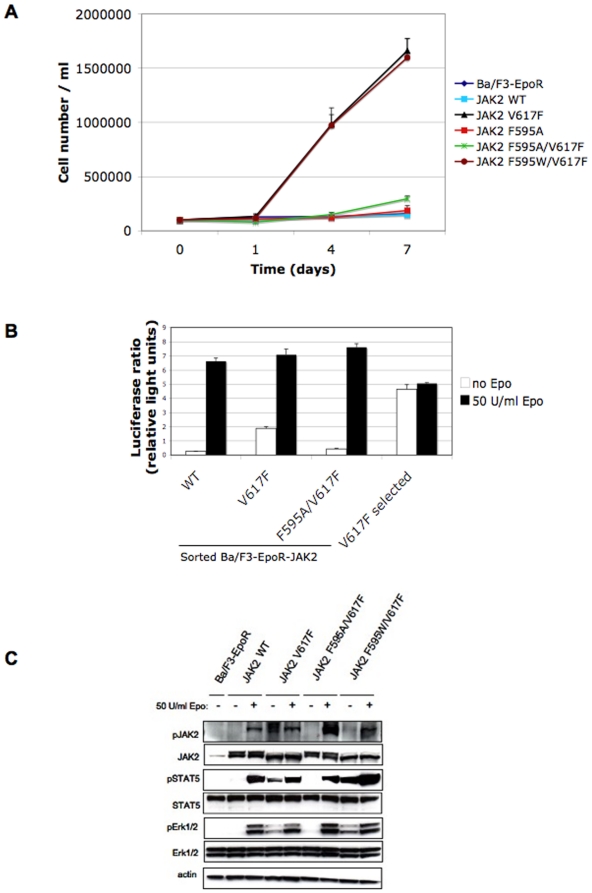
Role of JH2 αC helix residue F595 in the proliferation and activation of Ba/F3 cells stably expressing the Epo receptor and individual JAK2 mutants. (A) Proliferation assay of sorted Ba/F3-EpoR cells stably expressing each mutant and wild-type, in medium without growth factors for 7 days. JAK2 V617F is able to proliferate constitutively in this minimal medium starting from the first day (black triangles). The F595A/V617F double mutant lost most of its proliferative advantage (green stars), while the Ba/F3-EpoR parental cell line, or expressing either wild-type or F595A alone could not proliferate in the absence of growth factors. Substitution of F595 to an aromatic residue (F595W) restored autonomous growth (red circles). (B) Luciferase assay in sorted Ba/F3 cells stably expressing the Epo receptor and each individual JAK2 mutant or wild-type. Each cell line was individually electroporated with the pGRR5-Luc and pRLTK-Luc reporters and the STAT5 transcriptional activity was measured. The Ba/F3-EpoR cells expressing JAK2 V617F previously selected for autonomous growth show a typical increase in STAT5 transcriptional activity both in the presence and absence of Epo. (C) Immunoblot analysis of sorted Ba/F3-EpoR cells expressing individual mutants or wild-type, in presence of absence of Epo stimulation. pJAK2, pSTAT5 and pErk1/2 levels of JAK2 V617F, probed with specific antibodies revealed a constitutive signal in the absence of stimulation. This signal was absent in the cells expressing F595A/V617F but partially rescued in cells expressing F595W/V617F.

We subsequently investigated the effect of the F595 substitution on the constitutive activity of JAK2 V617F, in stably-expressing Ba/F3 cells and compared its activity level to the V617F single mutant and to wild-type. Ba/F3 are murine bone-marrow derived proB cells which depend on interleukin-3 (IL-3) for proliferation. Withdrawal of IL-3 leads to cell death, unless JAK2 V617F is expressed. We chose the F595 to Ala mutation as it induced a large decrease in the constitutive activity of V617F (Figure 2A). 293T-derived BOSC cells were used to produce retroviral supernatants of the JAK2 mutants cloned into the bicistronic vector pMX-IRES-GFP. Ba/F3 cells expressing the murine EpoR were infected with the supernatants and sorted for similar GFP levels 72 hours later. The sorted cells stably expressing each JAK2 mutant were washed and their proliferation in growth factor-free medium was followed for 7 days. Parental Ba/F3-EpoR cells and the sorted cells expressing JAK2 wild-type or JAK2 F595A were not able to support constitutive proliferation in this minimal medium, but could grow in the presence of Epo (data not shown), while JAK2 V617F-expressing cells proliferated at a very high rate in the absence of any growth factors (Figure 3A). The cells expressing JAK2 F595A/V617F lost most of their proliferative advantage, consistent with the presence of F595 being key to the preservation of constitutive activity (Figure 3A). As an additional control, we included Ba/F3-EpoR cells expressing JAK2 F595W/V617F, a mutant which maintained a level of constitutive activity almost identical to V617F in luciferase assays (Figure 2A). As expected, the JAK2 F595W/V617F mutant could also support autonomous growth of sorted Ba/F3-EpoR cells to a level identical to V617F (Figure 3A), pointing to an aromaticity requirement at position 595 playing a role in the constitutive activity of V617F.

Aliquots from the sorted cells expressing JAK2 wild-type, V617F and F595A/V617F were separately washed, incubated without serum and cytokines overnight (starvation) and electroporated with pGRR5-Luc and pRLTK-Luc reporters the next day. The STAT5 transcriptional activity induced by each mutant was measured two hours later. Similar to the γ-2A cells (Figure 2A), we observed a marked decrease in STAT5 transcriptional activity of the sorted Ba/F3-EpoR cells expressing the F595A/V617F mutant as compared to cells expressing V617F alone (Figure 3B). Ba/F3-EpoR cells expressing JAK2 wild-type displayed a low activity in the absence of ligand, but responded well upon stimutation with 50 U/ml Epo (Figure 3B). As an additional positive control, we included JAK2 V617F-expressing cells that had previously been selected for autonomous growth. As expected, these cells induced a constitutive STAT5 activation in the absence of cytokines almost double that induced by their sorted counterparts (Figure 3B).

Lastly, the sorted Ba/F3-EpoR cells expressing each mutant were stimulated or not with Epo, lysed and immunobloted in the presence of phosphospecific antibodies (Figure 3C). When comparing the pJAK2 and pSTAT5 levels in the total lysates, we were able to detect pJAK2 and pSTAT5 activities for V617F, in the absence of Epo stimulation. This activation was absent in the cells expressing JAK2 wild-type and JAK2 F595A/V617F, but could be detected to a lower degree in the cells expressing JAK2 F595W/V617F, consistent with the proliferation data (Figure 3A) and the transient transfection luciferase assay (Figure 2A). As expected, all mutants displayed strong pSTAT5 and pJAK2 activation upon Epo stimulation (Figure 3C). We also investigated how signaling via the Erk pathway was affected by the F595 mutations and detected that pErk1/2 levels were elevated in nonstimulated sorted cells expressing JAK2 V617F, but decreased once F595A mutation was additionally introduced. However, substitution of F595 to Trp, restored the autonomous pErk1/2 signaling (Figure 3C). Taken together, these results indicated that a Phe, or at least another aromatic residue, was required at position 595 to support JAK2 V617F constitutive signaling via the STAT and Erk pathways.

### Pseudokinase domain αC helix residue F595 is also required for constitutive activation of the exon 12 JAK2 K539L mutant and the JAK2 mutants T875N and R683G

We next asked the question whether the integrity of F595 also plays a role in the constitutive activity of other previously-described JAK2 mutants. In 3% of PV patients where the V617F mutation is not present, four somatic gain-of-function mutations in exon 12 of JAK2 have been reported, out of which JAK2 K539L, located in the linker region between the SH2 and JH2 domains, was the most common [28]. Another point mutation first identified in a megakaryoblastic cell line derived from an acute megakaryoblastic leukemia (AMKL) patient, and subsequently detected in a screen of human AMKL cell lines for STAT5 activation, presented a T875N substitution in the kinase domain of JAK2 [29]. The JAK2 R683G mutation located in the hinge region between N-terminal and C-terminal lobes of the pseudokinase domain, was identified in a screen of pediatric acute lymphoblastic leukemia (ALL) patient samples [30]. JAK2 K539L, T875N and R683G were shown to exhibit constitutive STAT5 activation and to transform Ba/F3 cells expressing the EpoR to cytokine-independence [28], [29], [30].

**Figure 4:**
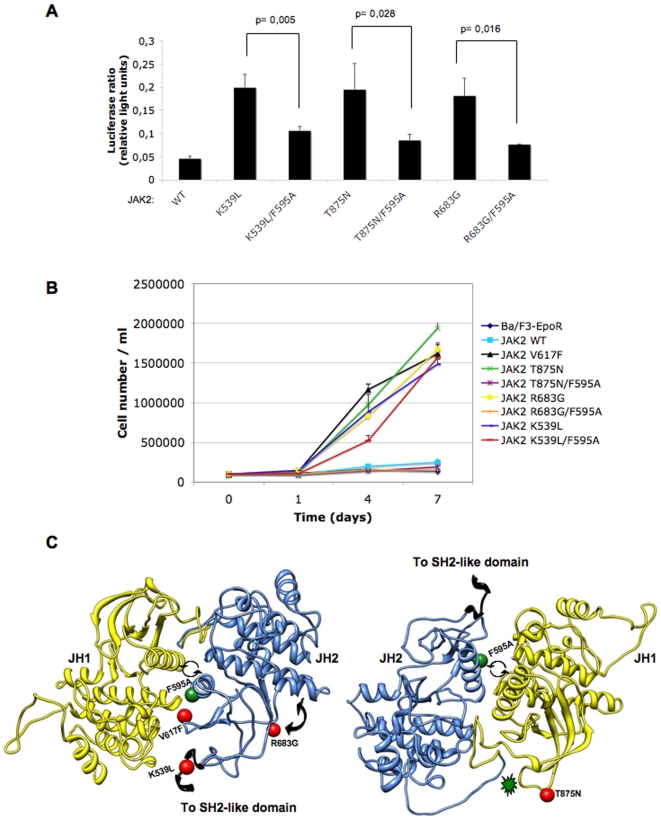
Substitution of F595 to Ala has an inhibitory effect on the constitutive activity of other JAK2 oncogenic mutants. (A) JAK2 K539L, T875N and R683G all induce constitutive activation of JAK2, however all three mutants display a marked decrease in STAT5 transcriptional activity in γ-2A cells when the F595A mutation is also introduced. (B) Proliferation assay of sorted Ba/F3-EpoR cells stably expressing each constitutive mutant and wild-type, in medium without growth factors for 7 days. Cells expressing JAK2 V617F (black triangles), K539L (blue lines), T875N (green stars) and R683G (yellow circles) can proliferate constitutively in this minimal medium starting from the first day. Substitution of F595 to Ala individually in the context of each active mutant causes a marked decrease in autonomous growth. The JAK2 K539L/F595A mutant (red lines) lost most of its proliferative advantage during the first 4 days, but by day 7 had regained the same level of autonomous growth as its single counterpart (blue lines). (C) Predicted locations (red spheres) of mutants K539L, R683G, V617F (left panel) and T875N (right panel) within the model structure of JAK2, based on coordinates of the homology model proposed by Lindauer *et al* [19]. The right panel is rotated 180° relative to the left panel. The kinase domain is depicted in yellow and the pseudokinase domain in blue. The location of F595 is shown as a green sphere. Circular arrows placed between helices C of JH2 and JH1 indicate potential conformational changes originating in JH2 and transmitted to JH1. Solid double-headed arrow placed near the R683G mutation suggests increased flexibility induced by this JH2 hinge mutant. Green star (right panel) suggests T875N mutation changes JH1-JH2 linker segment conformation, which is transmitted to JH2 helix C F595.

We introduced the F595A mutation individually in the context of JAK2 K539L, JAK2 T875N and JAK2 R683G and studied its effect on the activity of each mutant by quantifying the STAT5 levels in a luciferase assay of transiently transfected γ-2A cells. In all three mutants, JAK2 K539L, T875N and R683G, and similar to JAK2 V617F, the STAT5 activity was strongly inhibited by the presence of the F595A mutation (Figure 4A), indicating that F595 is also required for constitutive activation of these JAK2 mutants.

The signaling effects of the F595A mutation were also examined in sorted Ba/F3-EpoR cells expressing JAK2 K539L/F595A, JAK2 T875N/F595A and JAK2 R683G/F595A. Cells were washed and maintained in medium free of growth factors for 7 days. We observed that Ba/F3-EpoR cells expressing JAK2 V617F, JAK2 K539L, JAK2 T875N and JAK2 R683G could proliferate in this medium, while the same mutants with the additional F595A mutation lost most of their autonomous growth (Figure 4B). In the case of JAK2 K539L/F595A, the presence of the F595A mutation induced a decrease in the initial proliferation rate, but by day 7, these cells had regained the same proliferation ability as JAK2 K539L (Figure 4B). These results suggested that residue F595 is also important in the constitutive signaling of JAK2 T875N and JAK2 R683G and initially to JAK2 K539L, implying that activation of catalysis by JAK mutations may be mediated by conformational changes in the JH2 αC helix.

Based on its predicted location, the K539L mutation may affect the conformation of the upper V617 loop which would in turn interact with F595 and induce a rotation or conformational change in the JH2 αC helix triggering constitutive activity (Figure 4C, left panel). In the absence of F595 (i.e. F595A), it is tempting to speculate that the loop-helix interaction may be altered, decreasing constitutive activity. R683G may increase the flexibility of the linker region between the two lobes of the pseudokinase domain [30] and in doing so could require a certain conformation of the JH2 αC, which is lost when F595A is present. T875N is located in the kinase domain of JAK2, in a loop predicted to be within interaction distance of the linker region between the kinase and pseudokinase domains (Figure 4C, right panel) and could induce constitutive activity of JAK2 by triggering a conformational change of the pseudokinase domain. Crosstalk between the αC helices of JH1 and JH2 may be necessary for this, and introducing the F595A mutation could potentially alter the local conformation of the JH2 αC, hindering this helix-helix communication.

### The JAK1 helix C residue F636 is also required for constitutive activity of JAK1 V658F

**Figure 5:**
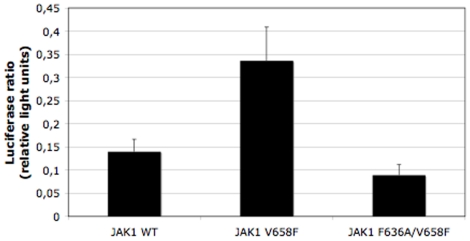
The F595A homolog in JAK1, F636A, blocks constitutive activity of JAK1 V658F. Transient transfection in the JAK1-deficient U4C cell line indicates a decrease in the STAT3 transcriptional activity in the JAK1 F636A/V658F, as compared to JAK1 V658F.

We have previously shown that the JAK2 V617F homologous mutation in JAK1, V658F, induces constitutive activation of JAK1 [31]. Recently the JAK1 V658F mutation was also identified from a screen of acute leukemia patients [32]. We now investigated whether the F595 homologous residue in JAK1, F636, also plays a role in the constitutive activation of this kinase, in a similar manner to JAK2. We substituted JAK1 residue F636 to Ala in the context of V658F, and quantified the STAT3 activity of this mutant in the JAK1-deficient cell line, U4C. As can be seen in Figure 5, substitution of F636 to Ala induces a large decrease in the constitutive activity of JAK1 V658F, indicating that the necessity of F636 is maintained in JAK1, similar to JAK2. This similar requirement of an intact F636 further points to the possibility that our data might also be relevant for JAK1 mutations recently identified in T-cell adult lymphoblastic leukemia (T-ALL) [32], [33].

### Orientation of the dimeric EpoR is essential for activation of JAK2 wild-type, but not for V617F

Given that JAKs are appended to the cytoplasmic domains of cytokine receptors, the hypothesis that V617F can induce activation of JAK2 in the absence of ligand through a specific interaction with the JH2 αC helix which triggers catalytic activity, has one immediate implication. That is, JAK2 V617F can overcome the requirement for a ligand-induced conformational change and effectively be capable of signaling from various relative conformations of a dimeric cytokine receptor, while in the wild-type scenario a ligand-induced conformational change in the receptor transmembrane and cytoplasmic domains is required, leading to the proper active conformation.

**Figure 6:**
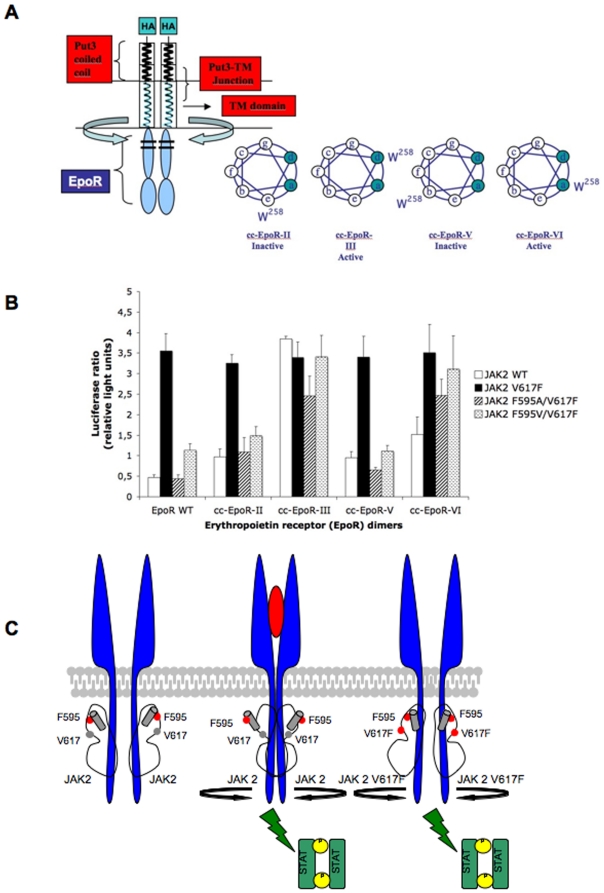
The JAK2 V617F mutation can induce constitutive activation regardless of the dimeric conformation of the receptor it is bound to, while JAK2 wild-type requires a ligand-activated EpoR dimer in order to signal. (A) The coiled-coil EpoR constructs were made by replacing the extracellular domain of EpoR with the dimeric coiled-coil domain of the yeast transcription factor Put3. (B) Luciferase assay in γ-2A cells transfected with each JAK2 and with previously engineered EpoR dimers containing coiled-coil replacements of their extracellular domains. JAK2 wild-type signals best from the EpoR dimeric conformations imposed by cc-EpoR-III and cc-EpoR-VI, previously shown to correspond to activated dimers. V617F has acquired the ability to signal constitutively from both active (cc-EpoR-III, cc-EpoR-VI) and inactive (cc-EpoR-II, cc-EpoR-V) dimeric interfaces. JAK2 F595A/V617F and F595V/V617F have lost the ability to signal constitutively from active and inactive dimeric conformations, suggesting that F595 may play a role in the constitutive activity of JAK2 V617F. Mutating F595 to Ala or Val forces the double mutant to prefer signaling from the active dimeric EpoR orientations (cc-EpoR-III and VI), akin to wild-type. (C) Activation model for cytokine-activated JAK2 versus JAK2 autoactivation caused by the V617F mutation. Only the pseudokinase domain is shown for simplicity. In wild-type JAK2, residues V617 (grey circle) and F595 (red circle in pseudokinase domain helix C, depicted as grey cylinder) do not play a key role in activation that is brought about by rotation of receptors and scissors-like movements, bringing JAK2 molecules in close proximity, and leading to activation of the kinase domain. In the case of the JAK2 V617F mutant, residues F617 (red circle) and F595 (red circle in pseudokinase domain helix C, depicted as a grey cylinder) are crucial for initiation activation, which is transmitted to the kinase domain of JAK2, in the absence of the rotation and scissors-like movement of the receptors.

In order to test whether this implication holds true, we employed a system we previously used to determine the active orientations of the transmembrane and cytosolic domains of the Epo receptor [34]. Briefly, the extracellular domain of EpoR was replaced with a dimeric coiled-coil and by varying the junction between the coiled coil and transmembrane domain, all seven possible conformations were imposed on the TM-cytosolic domains of EpoR (Figure 6A) [34]. The predictions of dimeric orientation were confirmed by cysteine-scanning mutagenesis of the transmembrane domain of EpoR in fusion with the Put3 coiled coil [35]. Using this system, we identified two conformations corresponding to an activated EpoR dimer (denoted cc-EpoR-III and cc-EpoR-VI) and two corresponding to an inactive EpoR dimer (denoted cc-EpoR-II and cc-EpoR-V) (Figure 6A) [34].

We now co-expressed these previously engineered active and inactive coiled-coil EpoR constructs with either JAK2 wild-type or V617F, along with STAT5, pGRR5-Luc and pRLTK-Luc reporters, and measured their STAT5 transcriptional activities in luciferase assays in γ-2A cells. We observed that, as expected, JAK2 wild-type signals best from the two conformations corresponding to an active EpoR dimer (III and VI) (Figure 6B). On other hand, consistent with our hypothesis, we observed that V617F was able to signal relatively well from both active (III and VI) and inactive (II and V) dimeric conformation (Figure 6B). This is in line with reports showing that presence of a dimeric receptor, like EpoR, supports constitutive activation of JAK2 V617F [36], [37].

We then compared the activities of two F595 mutants which decreased JAK2 V617F constitutive activity, F595A/V617F and F595V/V617F (Figure 2A) with V617F and wild-type in the same assay. We noticed that substituting the Phe at position 595, causes the F595A/V617F and F595V/V617F mutants to lose their ability to signal from both active and inactive dimeric conformations and to prefer the conformations corresponding to an activated EpoR dimer, similar to JAK2 wild-type (III and VI) (Figure 6B).

## Discussion

Our main observation is that residue F595, residing in the middle of the pseudokinase domain αC helix, plays a key role in the constitutive activation of JAK2 V617F. At the same time, the integrity of F595 was also key to several other JAK2 mutants located in different locations of the protein, JAK2 K539L, JAK2 T875N and JAK2 R683G. We present evidence that residue F595 is not crucial for the mechanism of ligand-induced JAK2 activation, as shown by the signaling abilities of the F595 single and double mutants in the presence of various concentrations of Epo ligand. Also, mutation of F595 to several different residues did not induce constitutive signaling from wild-type JAK2.

Mutation of F595 to Ala, Val, Ile or Lys in the context of V617F drastically decreased the constitutive activity of the V617F mutant in both the JAK2-deficient γ-2A and the hematopoietic Ba/F3 cell line, in transient and stable expression experiments, respectively. On the contrary, when F595 was replaced by an aromatic residue (Tyr or Trp) in the context of V617F, the constitutive activity was maintained. The substitution of F595 to Ala decreased the constitutive activity of JAK2 K539L, JAK2 T875N, and also of JAK2 R683G. Furthermore, constitutive activity of a JAK1 mutant homologous to JAK2 V617F (JAK1 V658F) was also inhibited by the homologous JAK1 F595 mutation (F636A), suggesting that our results in JAK2 might be relevant for activation of JAK1 mutants recently identified in T-ALL [32], [33].

A theoretical study was recently published [38], where molecular dynamics simulations indicated that F595 might be responsible for maintaining wild-type JAK2 inactive and, as a consequence, its possible interaction with F617 could alleviate this inhibition and contribute to activation. One the one hand, we agree with Lee *et al* that F595 is crucial for JAK2 V617F activity, but our results support the contrary notion for the wild-type JAK2, that is, F595 does not play a major role in the physiologic ligand-activation of JAK2, or in its inactivity in the basal state (Figures 2B and 6C). On the other hand, we do support a key role for F595 not only in the constitutive activity of JAK2 V617F, but also of several JAK2 oncogenic mutants.

Our data suggest that a specific interaction might occur between F595 in the JH2 αC helix and F617, predicted to be located in the loop between β-strands 4 and 5 (Figure 1C) [19]. We acknowledge that only a crystal structure of a complex of JAK2 JH1 and JH2 domains would decisively establish the presence of this interaction. In the case of JAK2 V617F, a Phe, Trp or Tyr at position 595 are able to maintain constitutive activity, suggesting an aromatic requirement at this position, and pointing to a possible specific stacking interaction between residues F595 and F617 as the most productive manner to activate the kinase domain. In the absence of ligand, such an aromatic initial interaction could induce a conformational change in the JH2 αC helix and trigger catalytic activity via repositioning of key catalytic residues. While this might represent the most efficient path to autoactivation, other, possibly less efficient paths must exist, that similarly rely on F595, since other JAK2 V617 mutations are also constitutively active (i.e. V617M, V617L, V617I [26]), and since the F595A mutation inhibits their activation (data not shown).

Given this scenario, we expected that when appended to a dimeric cytokine receptor, JAK2 V617F would signal irrespective of the particular dimeric orientation of the receptor, and that other less active mutants (V617M, V617L, V617I) might still depend on a conformational change of a dimeric receptor. This was indeed the case. When co-expressed with both active and inactive EpoR dimers, JAK2 V617F was able to induce a constitutive signal, regardless of the relative dimeric conformation (Figure 6B). JAK2 mutants V617I, V617L or V617M could signal from both inactive and active cc-EpoR dimer conformations, but they signaled stronger from active cc-EpoR dimers (data not shown). JAK2 wild-type, on the other hand, was activated only in the presence of the particular conformations corresponding to an activated EpoR dimer. Taken together, these data indicate that the F617: F595 pair is optimal for constitutive activation, irrespective of receptor conformation, but that other bulky aliphatic residues at 617 can induce constitutive activation of JAK2 via F595, with the caveat that such mutants might additionally require a conformational change of the scaffold receptor, in this case EpoR. In any case, F595 is pivotal for initiating autoactivation of JAK2, while it is not crucial for cytokine-induced JAK2 activation. In the wild-type scenario, a large conformational change of the receptor, that involves rotation [34] and a scissors-like movement [39], brings about kinase domain activation (model, Figure 6C).

Our data also provide evidence that pseudokinase domains might be pivotal to triggering kinase domain activation, even when mutations are not located in the proximity of pseudokinase domain residue V617, as is the case with JAK2 K539L (SH2-JH2 linker), JAK2 T875N (JH1) and JAK2 R683G (hinge of JH2) (Figure 4C). That pseudokinase domains appear in certain proteins to assume “active” conformations [40], [41] suggests that they evolved roles in transmission of conformational changes, and thus could be targets for inhibitors.

Several JAK2 kinase domain inhibitors, that are ATP-binding competitors are in clinical trials for primary or secondary myelofibrosis [42], [43]. These inhibitors do not discriminate between wild-type and mutant JAK2, and can induce unwanted effects, such as anemia and thrombocytopenia. An ideal inhibitor for patients harboring JAK mutants would have to preferentially target the mutant JAK and spare signaling by the wild-type JAK. We suggest that the region in the middle of the pseudokinase domain helix C around residue F595 could be the target of such a specific inhibitor.

## Materials and Methods

### JAK2 mutant plasmid constructs

All murine JAK2 (and JAK1) mutants were obtained by PCR reactions with mutagenic complementary forward and reverse primers by the QuickChange Site-Directed Mutagenesis method (Stratagene). All constructs were cloned into the bicistronic retroviral vector pMX-IRES-GFP and verified by sequencing. In most cases, 2 independent clones were tested per each mutant.

### Cell lines and retroviral transductions

γ-2A and U4C are JAK2-deficient and JAK1-deficient human fibrosarcoma cells [27], [44]. Ba/F3-EpoR cells are murine IL-3-dependent cells that are expressing the murine erythropoietin receptor. Wild-type and mutant JAKs were transfected into BOSC packaging cells to produce retroviruses which were subsequently used to infect Ba/F3-EpoR cells as described [34]. GFP positive cells were sorted by FACS 72 hours after infection, washed and cultured in the absence of cytokines in RPMI medium + 10% FBS. Cell numbers were recorded over a period of seven days with a Coulter cell counter. The V617F mutant which acquired the ability to proliferate in the absence of cytokines was further cultured in RPMI medium + 10% FBS. The sorted Ba/F3-EpoR cells expressing the JAK2 mutants or wild-type were maintained in RPMI medium + 10% fetal bovine serum and WEHI cell supernatant, as a source of IL-3.

### Dual luciferase assays

The STAT5 transcriptional activity of the various mutants was measured in γ-2A cells (fibrosarcoma cells deficient in JAK2) by dual luciferase assays with the STAT reporter, pGRR5-Luc [45]. Cells were seeded in 24-well plates overnight and transfected using lipofectamine, with pGRR5-Luc, STAT5, EpoR, the cDNA coding for each individual JAK mutant and pRLTK-Luc as an internal control. The STAT3 transcriptional activity of JAK1 mutants was measured in JAK1-deficient U4C cells [44] by transiently transfecting each JAK1 construct, the pGRR5-Luc and pRLTK-Luc reporters and the interleukin-9 receptor (IL-9R). Medium was changed 4 hours after transfection and stimulation with Epo was performed when stated. The cells were lysed 24 hours after transfection and luminescence was recorded on a TD-20/20 or Glomax 96-well plate luminometer. When performing the assay on stably transduced sorted Ba/F3-EpoR cells (expressing each JAK2 mutant), cells were starved overnight in RPMI medium with 1 mg/ml BSA, next day stimulated or not with 50 U/ml Epo and electroporated with the pGRR5-Luc and pRLTK-Luc luciferase reporters. The cells were subsequently cultured for 2 hours, lysed in 100 µl 1X passive lysis buffer and their luminescence was recorded.

### Immunoblotting

2.5×10^7^ Ba/F3-EpoR cells expressing each JAK2 mutant were starved overnight in RPMI medium with 1 mg/ml BSA, and stimulated or not with 50 U/ml Epo for 15 minutes. Cells were resuspended in cold lysis buffer (1%NP40 + 1X Protease Inhibitor Cocktail (Roche), 1 mM vanadate, 1 mM PMSF). Upon incubation on ice for 30 minutes and spinning for 20 minutes at 20,000 g and 4°C, the supernatant was mixed with an appropriate volume of 2X Laemmli, boiled and 40 µg total protein was loaded on NuPage 4–12% Bis-Tris gels (Invitrogen). Transfer on nitrocellulose membranes was carried out with the iBlot™ Dry Blotting System (Invitrogen).

Western blotting antibodies were directed against: phospho-JAK2 (Millipore), JAK2 (Santa Cruz), beta-actin (Sigma), and phospho-STAT5 A/B (Tyr694), phospho-Erk1/2 (Tyr202/Tyr204), Erk1/2 (Cell Signaling Technology).

### Structural modeling

The coordinates for the homology model of JAK2 in an inactive conformation were obtained from Dr. Romano Kroemer. PDB coordinates for JAK2 JH1 active structure were obtained from the PDB database (Accession codes 2B7A, 3FUP). All other PDB coordinates were obtained from the PDB database as described in each case. Molecular graphics images were produced using the UCSF Chimera package from the Resource for Biocomputing, Visualization, and Informatics at the University of California, San Francisco (supported by NIH P41 RR-01081) [46].

## Supporting Information

Figure S1 Mutations in the beginning segment of the JH2 αC helix do not alter the constitutive activity of JAK2 V617F. Substitution of JH2 helix C residues R588 and E592 simultaneously to Ala has no effect on the STAT5 transcriptional activity of JAK2 V617F (or JAK2 wild-type as a control), unless F595 is also mutated.
(0.06 MB TIF)
