# Supplementary figures and images for: JAK2 V617F Constitutive Activation Requires JH2 Residue F595: A Pseudokinase Domain Target for Specific Inhibitors
Source: PLoS One. 2010 Jun 16;5(6):e11157. doi: 10.1371/journal.pone.0011157 (PMC2886835; doi:10.1371/journal.pone.0011157)

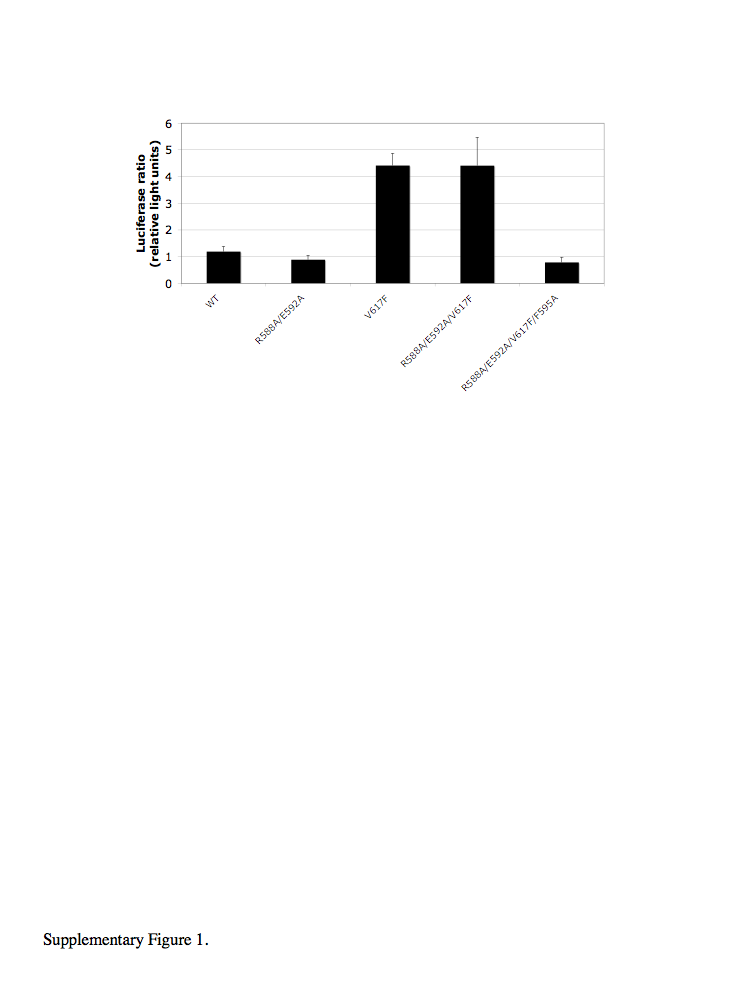

Supplement: Figure S1 — Mutations in the beginning segment of the JH2 αC helix do not alter the constitutive activity of JAK2 V617F. Substitution of JH2 helix C residues R588 and E592 simultaneously to Ala has no effect on the STAT5 transcriptional activity of JAK2 V617F (or JAK2 wild-type as a control), unless F595 is also mutated. (0.06 MB TIF) [file pone.0011157.s001.tif]
